# Experimental and Numerical Simulation Studies on V-Shaped Bending of Aluminum/CFRP Laminates

**DOI:** 10.3390/ma16144939

**Published:** 2023-07-11

**Authors:** Hang Cheng, Zhiqiang Zhang, Mingwen Ren, Hongjie Jia

**Affiliations:** Key Laboratory of Automobile Materials, Ministry of Education, School of Material Science and Engineering, Jilin University, Changchun 130022, China; chenghang20@mails.jlu.edu.cn (H.C.); renmw@jlu.edu.cn (M.R.); jiahj@jlu.edu.cn (H.J.)

**Keywords:** fiber metal laminate, bending, springback, damage, numerical simulation

## Abstract

With the increasing requirements of automotive lightweighting, metal/CFRP laminates are increasingly used. In this paper, Al/CFRP laminates were prepared using an integrated hot press curing method, and the optimum curing conditions were determined using the single-lap shear test at 130 °C for 45 min. The effects of fiber lay-up, forming speed, and metal layer thickness on bending springback were investigated using the V-shaped bending test and Abaqus finite element analysis method. The results show that fiber lay-up has an important influence on springback. Among the five different fiber lay-ups (0° unidirectional, 90° unidirectional, 0° orthotropic, 90° orthotropic, and 45° orthotropic), the 45° orthotropic lay-up had the lowest springback rate of 1.11%. Increasing the thickness of the sheet metal can significantly reduce the resilience rate. As the sheet thickness increased from 2 mm to 3 mm, the springback of the 90° unidirectional lay-up decreased by 43%. Springback was not sensitive to forming speed, and the difference in springback was within 1% at different forming speeds. The damage behavior of the forming process was analyzed using the three-dimensional Hashin damage law with the Vumat subroutine and microscopic analysis. Fiber and resin damage under 45° orthotropic lay-up conditions was relatively small compared to fiber damage under 0° unidirectional lay-up and resin damage under 90° unidirectional lay-up.

## 1. Introduction

Along with the increasing production of automobiles, the corresponding energy consumption and environmental protection problems are becoming increasingly serious. Energy savings and emission reduction have become the primary issues that need to be addressed for the sustainable development of the automotive industry. Vehicle weight reduction is one of the effective measures to improve energy efficiency and reduce emissions [1]. Carbon fiber reinforced plastics (CFRP), which exhibit a high specific modulus and high designability, have great potential for automotive lightweighting [2,3,4,5]. However, the poor plasticity and fracture toughness of CFRP materials make it possible to produce sharp corners after damage fracture, causing damage to passengers [6]. CFRP/metal composite laminates [7,8,9] can effectively reduce the cost of production, without compromising strength and stiffness, while overcoming the shortcomings of single material performance [10,11]. With the development of automotive lightweighting, 7000 series aluminum alloy materials have gradually replaced traditional steel materials [12,13,14,15,16]. A composite of 7000 series aluminum alloy and CFRP material can obtain excellent comprehensive performance of the high strength of CFRP and the good toughness of aluminum alloy.

For Al/CFRP composite laminates, common processing methods include resin transfer molding [17], autoclave molding [18], and hot press curing integrated molding. Resin transfer molding is prone to defects, such as inadequate curing of CFRP and uneven thickness after molding. Autoclave molding has a high cost and high requirements for temperature and vacuum, and poor temperature control accuracy cannot make complex shape parts.

Hot press curing integration has the advantages of low cost and short production cycle. It first heats the forming dies and then forms and cures the metal/CFRP hybrid part in one stroke using epoxy resin in CFRP as the adhesive. Uriya et al. [19] investigated the springback of V-shaped CFRP/Al hybrid parts at different temperatures. Wang et al. [20] formed Al/CFRP laminates and studied the effect of forming temperature on formability. Tanaka et al. [21] used CFRP containing thermoplastic epoxy resin, laminated with Al alloy sheets to form hat-shaped parts, and analyzed the lay-up sequence of CFRP, the material thickness ratio of Al and CFRP, and the effect of the stacking method on stiffness using the finite element method. The model using CFRP with a stacking sequence of (±45) showed higher stiffness. Hu et al. [22] investigated the mechanical properties and failure mechanisms of CFRP/Al laminates. The bending behavior under different stacking sequences was investigated using three-point bending tests. The results suggest that the carbon fiber reinforced composite layers played a prominent role in the bending properties. With an increasing volume fraction of aluminum sheets, lower bending strength and modulus were obtained for the laminates. Different fiber orientations have different effects on forming damage. However, due to the complexity of the layer, material failure mechanism, and difficulty of forming, there is very limited research on the forming of CFRP/7000 aluminum alloy laminates, especially the springback after forming.

This paper focuses on the forming and springback study of V-bending of 7075 aluminum alloy/CFRP laminates in order to have a deep understanding and effective control of springback behavior. Forming accuracy was measured based on the amount of springback. The experiments were performed using a V-bending mold, and the simulations were performed using Abaqus/Explicit. Forming damage, such as fiber fracture and matrix fracture, was predicted using the subroutine Vumat.

## 2. Experimental Methods and Materials

The CFRP was T700 carbon fiber prepreg produced by Toray, Tokyo, Japan, with a thickness of 0.15 mm. R5600 standard prepreg resin with 38% resin content was used. The T700 material properties are shown in Table 1 [23]. E1 is the longitudinal modulus of elasticity, E2 is the transverse modulus of elasticity, μ is Poisson’s ratio, and G is the shear modulus. Aluminum sheets (7075-T6) of 2 mm and 3 mm were selected, and the properties are shown in Table 2.

### 2.1. Preparation of Composite Laminates

**Figure 1:**
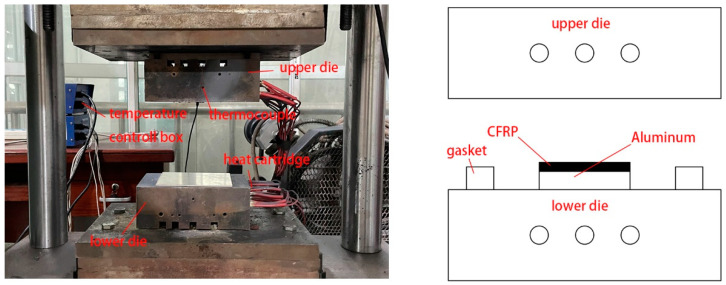
Hot pressing curing integrated set-up.

The composite laminates were prepared using the hot press curing integrated method. The hot press curing device is shown in Figure 1, which includes an upper die, a lower die, heat cartridges, a thermocouple, and a temperature control box. The heating box has a power of 1 kW and a temperature control range of 0–999 degrees Celsius. First, the aluminum alloy sheet surface was sanded with 80-grit sandpaper and ultrasonically cleaned to remove debris. Then, 6 layers of CFRP were manually laid on the treated aluminum sheet surface. When the temperature reached the curing temperature (120–140 °C), the composite laminate would be placed between the upper die and the lower die. The laminate was cured under pressure at 18 MPa for 30–60 minutes and then removed from the dies.

### 2.2. Determination of Curing Conditions

**Figure 2:**
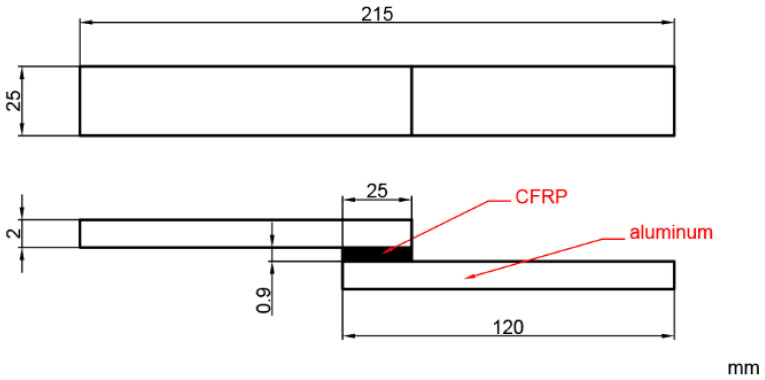
Specimen of single-lap shear test.

**Figure 3:**
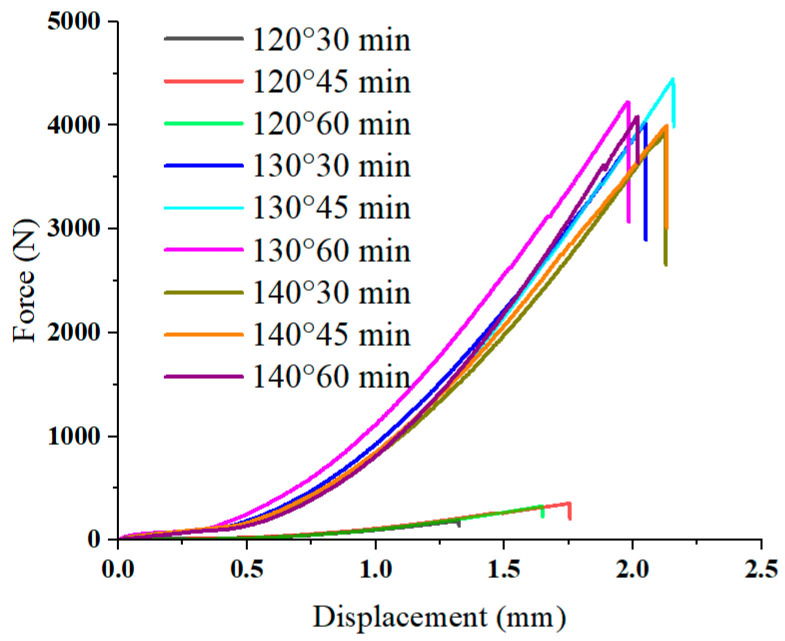
Force–displacement curves of single-lap joint shear tests.

**Figure 4:**
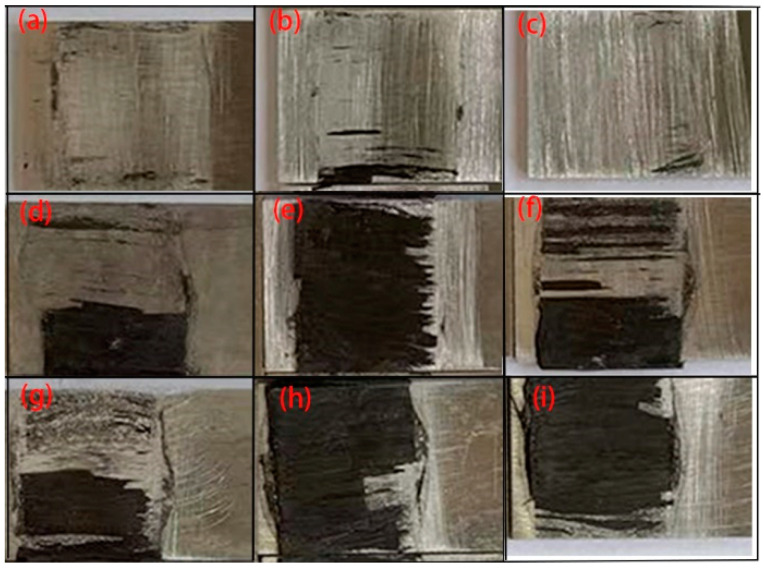
The macroscopic morphology of the interface after the shear test. (**a**) 120 °C-30 min; (**b**) 120 °C-45 min; (**c**) 120 °C-60 min; (**d**) 130 °C-30 min; (**e**) 130 °C-45 min; (**f**) 130 °C-60 min; (**g**) 140 °C-30 min; (**h**) 140 °C-45 min; (**i**) 140 °C-60 min.

The connection between the CFRP and the aluminum sheet relies on the epoxy resin in the CFRP prepreg. This resin starts to flow under heat, enters the pits in the rough metal surface, and starts to cure when the curing temperature is reached, thus achieving a tight connection between the CFRP and the aluminum sheet. Curing conditions have an important influence on the quality of the CFRP/aluminum alloy interface connection. The best curing conditions were determined using the single-lap shear test, using the interfacial bearing capacity as a measure. The single-lap shear specimen was designed according to ASTM-1002, as shown in Figure 2. The CFRP prepreg was laminated with 6 layers of 0° unidirectional lay-up. After placing the prepreg over the aluminum sheet, they were hot pressed. The specimens were subjected to the single-lap shear test using a WDW-20 electronic universal testing machine (KeXin, Changchun, China) with a loading speed of 2 mm/s. The average of two experimental results was taken for each group. The force–displacement curves of the experimental results are shown in Figure 3. The fracture morphology of the specimen is shown in Figure 4.

When curing at 120 °C, the interfacial bearing capacity was generally very low, showing an order of magnitude difference from the experimental results of the other groups. The interfacial bearing capacities of the 30 min group and 45 min group were approximately 0.17 kN and 0.30 kN, respectively. According to the morphology at the debonding of CFRP/Al laminate, CFRP completely separated from the Al sheet surface, with the surfaces smoother and the amount of residual fibers less, which was considered to be caused by incomplete curing. When the thermosetting resin is incompletely cured, the resin remaining on the rough metal surface does not create a sufficient connection between the CFRP and the metal sheet. Thus, when subjected to a load, the interface is damaged before the fibers and the CFRP separate from the aluminum. For the 130 °C curing group, the interface bearing capacity reached 4.01 kN at 30 min. When the curing time reached 45 min, the bearing capacity reached a peak of 4.40 kN. Compared to the 120 °C groups, the bearing capacity increased by 2258.82% and 1366.67%, respectively. A large amount of fiber residue was visible at the debonding surface, and the fiber bundles appeared to be fractured. The curing was thus considered completed, the interfacial strength was higher than the interlayer strength, and the bearing capacity was also greatly increased. When the curing time reached 60 min, there was a slight decrease in the bearing capacity due to the excessive curing time, which led to aging of the resin. It can be found that the bearing capacity of the 140 °C curing group did not exceed that of the 130 °C group. From both performance and benefit perspectives, 130 °C-45 min was chosen as the curing condition.

### 2.3. V-Shaped Bending Test

**Figure 5:**
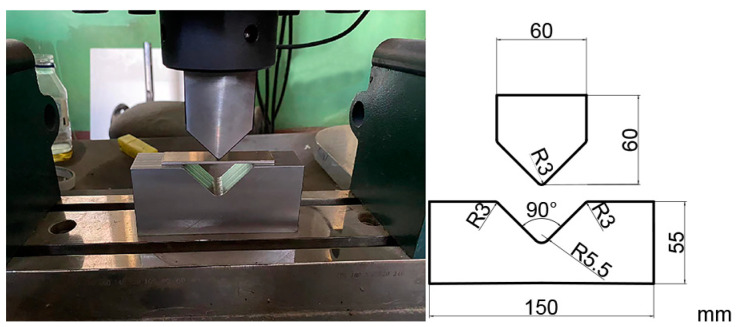
V-bending dies.

Due to the poor formability of 7075-T6 at room temperature, the aluminum alloy sheet was solution treated at 480 °C for 30 min and rapidly quenched before being laminated with CFRP. The size of the 7075 aluminum alloy sheet was 80 mm × 20 mm, and the thickness was 2 mm and 3 mm. The thickness of the single layer prepreg was 0.15 mm. Six layers of prepreg were laid in 0° unidirectional (0/0/0/0/0/0), 90° unidirectional (90/90/90/90/90/90), 45° orthotropic (45/−45/45/−45/45/−45), 90° orthotropic (0/90/90/90/90/0), and 0° orthotropic (90/0/0/0/0/90). After lay-up, the laminate was hot pressed and cured at 130 °C for 45 min. The bending dies and dimensions are shown in Figure 5. 

### 2.4. Springback Rate Measurement

**Figure 6:**
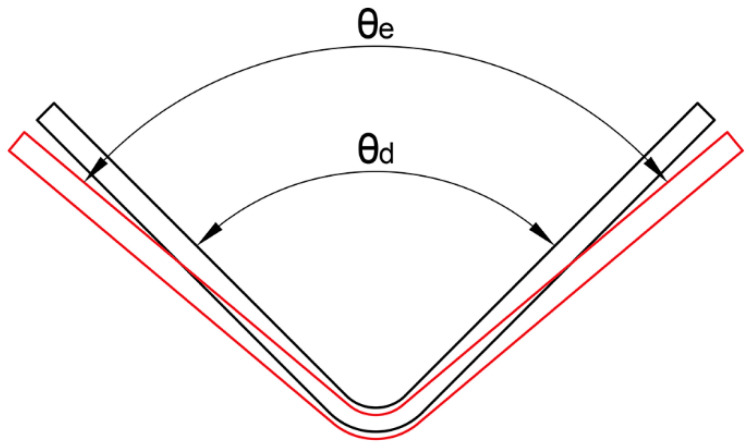
Springback after forming.

The angles of the unloaded parts were measured using a multifunctional protractor. The difference in angle before and after forming is shown in Figure 6.

Use the springback rate to characterize the degree of springback.

(1) $$K=\frac{\theta_{e}-\theta_{d}}{\theta_{d}}\times 100\%$$

where $\theta_{e}$ is the angle after springback, $\theta_{d}$ is the angle before springback, and K is the springback rate.

## 3. Results and Discussion

### 3.1. Effect of Lay-Up Direction on Springback

**Figure 7:**
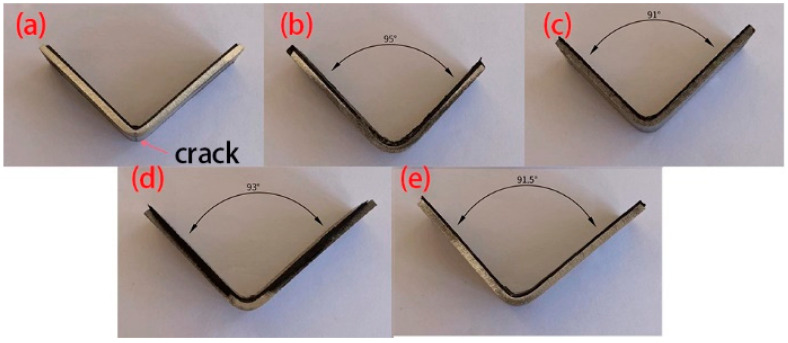
Bending parts with different layer-ups. (**a**) 0 unidirectional; (**b**) 90 unidirectional; (**c**) 45 orthotropic; (**d**) 90 orthotropic; (**e**) 0 orthotropic.

**Figure 8:**
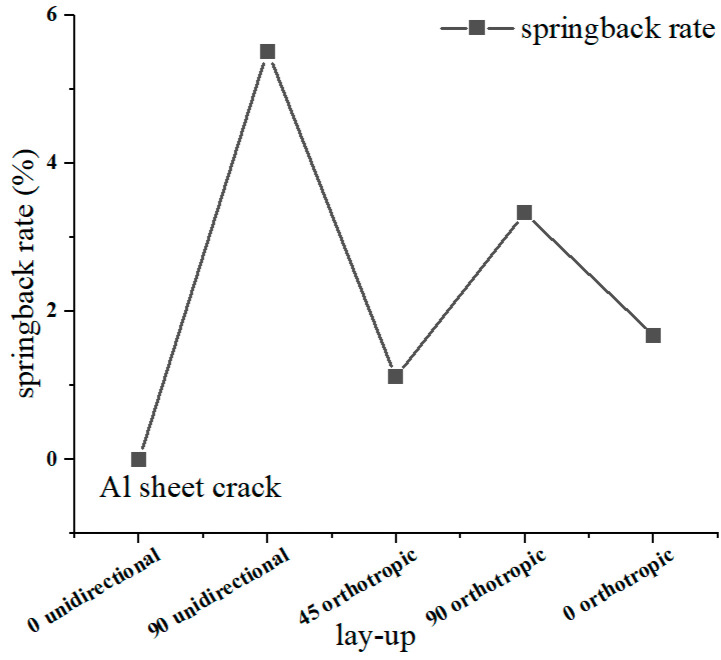
Springback rate on lay-up orientation.

The bending parts with different layer-up sequences are shown in Figure 7. The corresponding springback rates are shown in Figure 8, where the 90° unidirectional springback is 5.5%, the 45° orthotropic spingback is 1.11%, the 90° orthotropic springback is 3.33%, and the 0° orthotropic springback is 1.67%. It is obvious that lay-up direction has an important effect on springback, which is mainly reflected in the resistance to deformation of different lay-ups, i.e., the elastic modulus E. 

**Figure 9:**
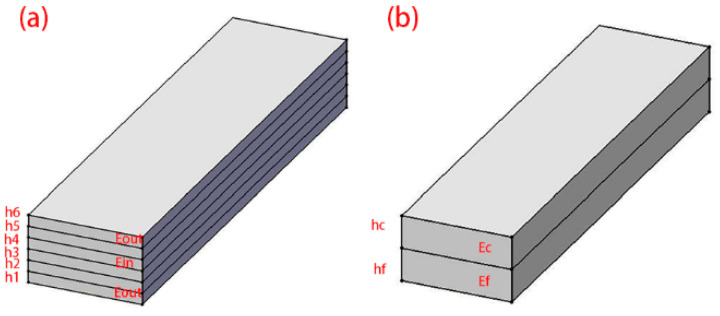
Simplified model. (**a**) CFRP; (**b**) CFRP/Al.

The simplified laminate models are shown in Figure 9. The equivalent elastic moduli of different lay-up laminates can be obtained using the composite theory. The elastic moduli of 0° unidirectional and 90° unidirectional are 130 GPa and 8 GPa, respectively. For the other lay-ups, the core layer thickness/total thickness ratio v is introduced.

(2) $$v=\frac{h_{2}+h_{3}+h_{4}+h_{5}}{h_{1}+h_{2}+h_{3}+h_{4}+h_{5}+h_{6}}$$

According to the classical compound rule, the equivalent elastic modulus $E_{c}$ is given by:

(3) $$E_{c}=E_{in}v+E_{\mathrm{out}\;}(1-v)$$

$E_{in}$ is the elastic modulus of the middle four layers, and $E_{\mathrm{out}}$ is the elastic modulus of the outer two layers.

The equivalent elastic moduli of the different lay-up laminates are shown in Table 3.

Use the same method to obtain the equivalent elastic modulus $E_{s}$ of the Al/CFRP hybrid laminates:

(4) $$E_{s}=E_{c}v_{c}+E_{f}(1-v_{c})$$

$E_{f}$ is the modulus of elasticity of the aluminum sheet, and $E_{s}$ is the effective modulus. The elastic modulus of the hybrid laminate is shown in Table 4. 

**Figure 10:**
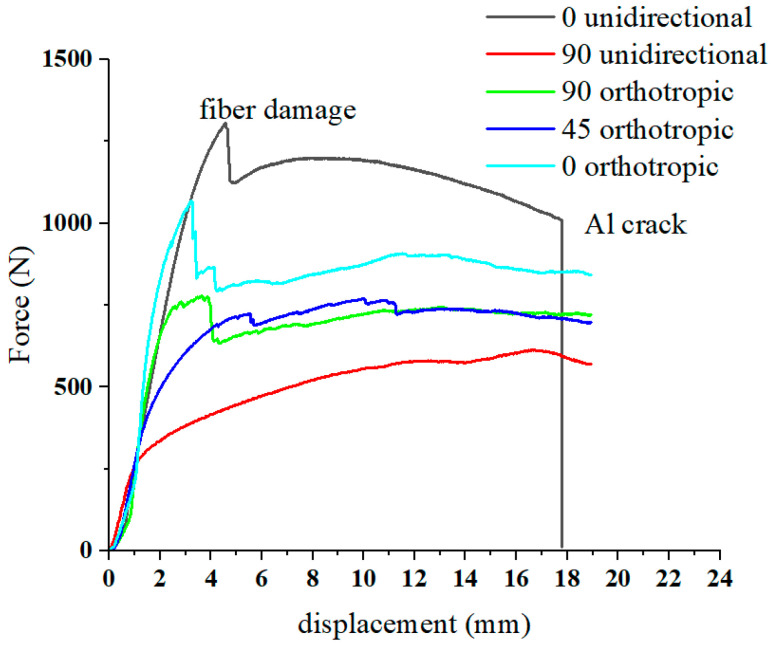
Bending characters of different lay-up laminates.

The force–displacement curves with different lay-up laminates are shown in Figure 10. For the laminate with 0° unidirectional lay-up, it was subjected to tensile stress during bending and had a high resistance to deformation. However, the fracture of the fibers and the aluminum alloy sheet occurred during the forming process. The force–displacement curve of the 0° unidirectional lay-up showed two abrupt drops. Because of the aluminum alloy sheet and fiber fracture, the release of residual stress leads to less springback. For the 90° unidirectional lay-up, the fiber bundle flows to both sides along the punch loading direction, and the resistance to deformation is weak. Although fiber fracture also occurs, the residual fiber and aluminum alloy sheet can promote springback. For the 45° orthotropic lay-up, the in-plane shear modulus is much smaller than its tensile modulus along the fiber direction; therefore, during the bending and forming process, the resistance to deformation is weak, causing less springback. Comparing the 90° orthotropic and 0° orthotropic lay-up, it can be seen that the more 0° lay-up, the smaller the springback. 

### 3.2. Influence of Forming Speed

**Figure 11:**
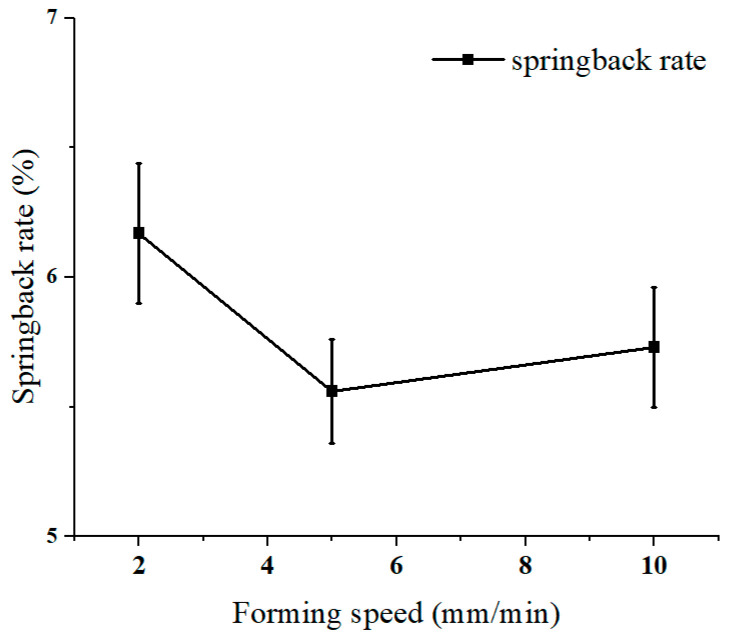
Springback rate with different forming speeds.

The effect of forming speed on the 90° unidirectional fiber lay-up laminate was studied. The forming speeds were 2 mm/s, 5 mm/s, and 10 mm/s. The springback rates are shown in Figure 11. The springback rates were 6.17% for 2 mm/s, 5.56% for 5 mm/s, and 5.73% for 10 mm/s. Forming speed had little effect on springback. On the one hand, the increase in forming speed enhances the hardening effect of the aluminum alloy, and thus the springback is more obvious. On the other hand, the increase in forming speed causes a thermal effect; part of the energy consumed in plastic deformation is converted into heat, which increases the temperature of the metal and improves the forming properties, which contributes to the reduction in springback.

### 3.3. Effect of Metal Thickness

**Figure 12:**
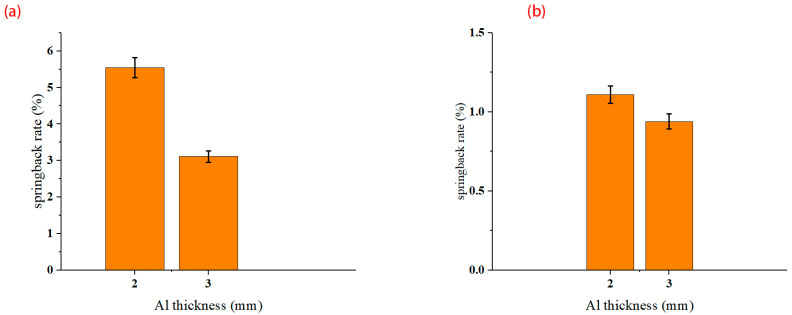
Influence of metal thickness. (**a**) 90° unidirectional; (**b**) 45° orthotropic.

Figure 12 shows the influence of different thicknesses of aluminum alloy sheets on springback. For the 90° unidirectional lay-up, the springback rate is 5.55% for the 2 mm aluminum alloy sheet and 3.11% for the 3 mm aluminum alloy sheet (Figure 12a). For the 45° orthotropic lay-up, it also shows that the springback rate will decrease with the increase in aluminum alloy sheet thickness. The effect of different metal thicknesses on laminate bending is mainly controlled by the relative bending radius. When r/t is small, the elastic deformation accounts for a low proportion, and the springback reduces.

## 4. Finite Element Analysis

### 4.1. Finite Element Modeling

**Figure 13:**
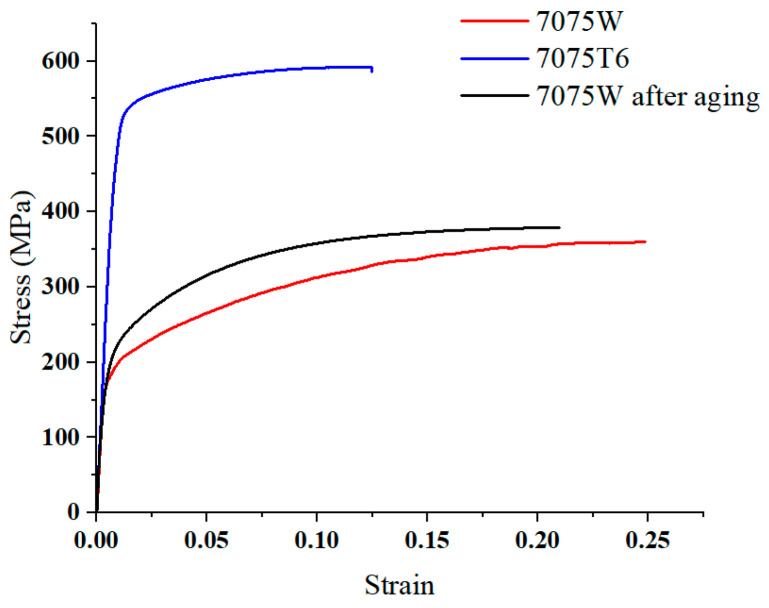
Stress–strain curves of 7075 aluminum.

**Figure 14:**
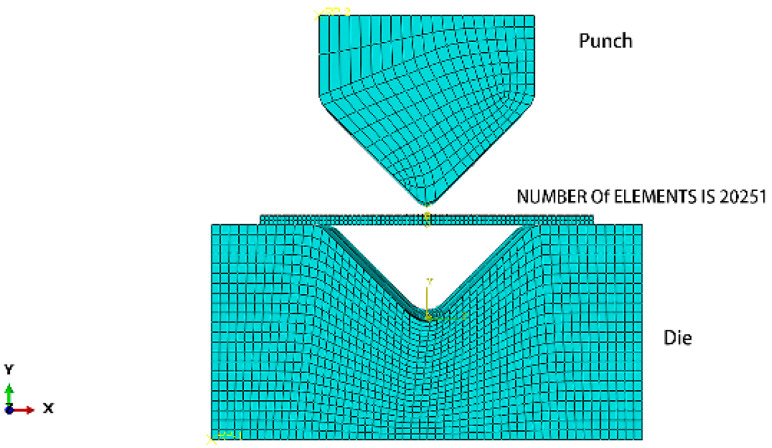
Mesh of V-bending set-up.

**Figure 15:**
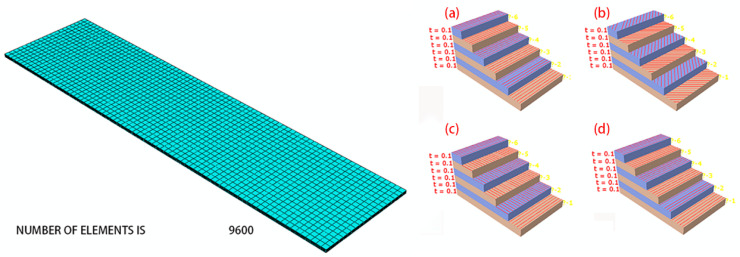
Mesh of lay-up. (**a**) 90 unidirectional; (**b**) 45 orthotropic; (**c**) 0 orthotropic; (**d**) 90 orthotropic.

Simulation analysis of bending and springback was performed using Abaqus/Explicit. This simulation method uses the central difference method to complete the simulation with the help of multiple time increments and is suitable for analysis with large deformations. The 7075-T6 aluminum alloy sheet was subjected to hot pressing treatment after solution, which is equivalent to aging treatment. The stress–strain curves with different heat treatments are shown in Figure 13. The solution treatment improved the plasticity of the 7075 aluminum alloy and reduced the strength, but the W-state aluminum alloy was in an unstable state. After hot pressing at 130 °C for 45 minutes, the properties of the material tended to be stable, and the plasticity was greatly improved compared to that of the T6 state, which can be used for bending forming. The mechanical properties of the 7075 aluminum alloy sheet after hot pressing at 130 °C for 45 minutes are shown in Table 5. The mesh model is shown in Figure 14. The aluminum alloy sheet is C3D8R with a mesh size of 0.2 mm, and CFRP is C3D8R with a mesh size of 0.1 mm. Theoretically, the smaller the meshing size, the more realistic the simulation results; however, increasing the number of meshes can significantly increase the computing time and may cause the software to crash. For this consideration, meshing was performed. The punch and die are non-deformed rigid cells, and the mesh shape is quadrilateral with a neutral axis algorithm. The lay-up of CFRP modeling is shown in Figure 15.

The resin in CFRP acts as an adhesive, so the interface between the CFRP layers and between Al/CFRP was defined as cohesive contact. To observe whether the interface failed to separate, the damage criterion was the traction rule. In order to converge the simulation results, the viscosity coefficient was defined as 0.0001 (large coefficients can lead to scattered results and cause simulation errors). To avoid mesh intrusion and uncontrolled slippage, the overall contact was defined as normal hard contact and tangential penalty contact, and the friction coefficient was 0.25. To reduce the calculation time, the step was set up with mass scaling, while the time step was reduced to 0.01 s. The load of the springback was set to a predefined field, and the odb file from the previous step was imported for stress relief.

Based on the three-dimensional Hashin damage criterion, damage behavior was simulated using a user-defined subroutine Vumat. The solution-dependent state variables (which change with the increase in time step) SDV1, SDV2, SDV3, and SDV4 corresponded to fiber tensile damage, fiber compression damage, matrix tensile damage, and matrix compression damage, respectively.

3D Hashin damage law [24].

To fiber damage

(5) $$\sigma_{11}>0{\left(\frac{\sigma_{11}}{x_{T}}\right)}^{2}+{\left(\frac{\sigma_{12}}{s_{12}^{2}}\right)}^{2}+{\left(\frac{\sigma_{13}}{s_{13}^{2}}\right)}^{2}\geq 1$$

(6) $$\sigma_{11}<0{\left(\frac{\sigma_{11}}{x_{C}}\right)}^{2}\geq 1$$

To matrix damage

(7) $$\sigma_{22}>0{\left(\frac{\sigma_{22}}{Y_{T}}\right)}^{2}+{\left(\frac{\sigma_{12}}{s_{12}^{2}}\right)}^{2}+{\left(\frac{\sigma_{23}}{s_{23}^{2}}\right)}^{2}\geq 1$$

(8) $$\sigma_{22}<0{\left(\frac{\sigma_{22}}{Y_{C}}\right)}^{2}+{\left(\frac{\sigma_{12}}{s_{12}^{2}}\right)}^{2}+{\left(\frac{\sigma_{23}}{s_{23}^{2}}\right)}^{2}\geq 1$$

where σ represents Cauchy stress, and X_T_ and X_C_ represent the tensile and compressive strengths in the fiber direction, respectively. Y_T_ and Y_C_ refer to the tensile and compressive strengths in the matrix direction, and Z_T_ represents the tensile strain strength in the fiber thickness direction. S_12_, S_13_, and S_23_ represent the in-plane and out-of-plane shear strains.

### 4.2. Simulation Results 

**Figure 16:**
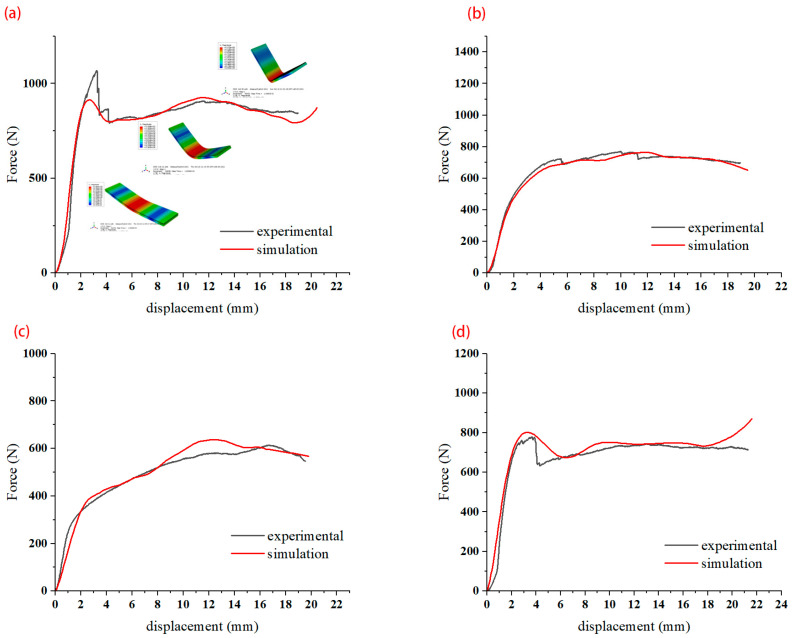
Simulation and experimental comparison of force–displacement. (**a**) 0 orthotropic; (**b**) 45 orthotropic; (**c**) 90 unidirectional; (**d**) 90 orthotropic.

**Figure 17:**
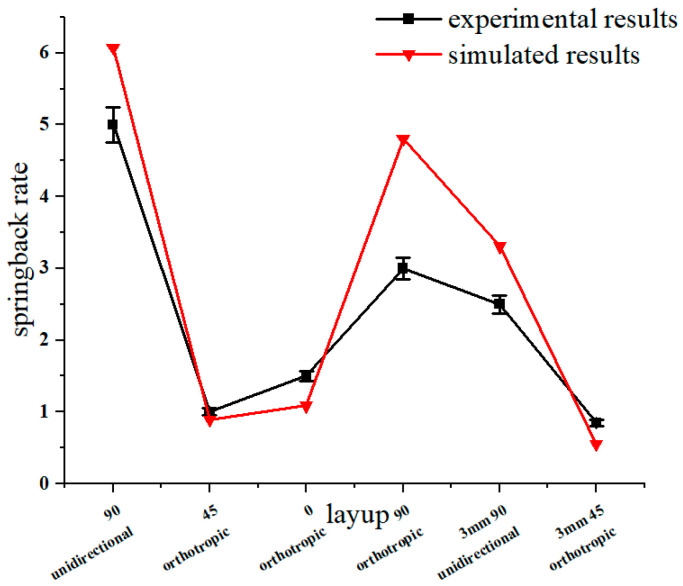
Simulation and experimental results of springback.

A comparison of the simulated force–displacement curves and the experimental force–displacement curves is shown in Figure 16. When forming begins, the curve rises rapidly in the elastic phase, drops after encountering varying degrees of fiber damage, and enters the plastic deformation phase; due to the closure of the mold, the load rises after final forming. Partial fracture of the fibers during bending leads to a drop in the curve. In each stage of bending, the experimental and simulated curves can be seen to be in general agreement, which verifies that the proposed model is more consistent. The springback simulation results are shown in Figure 17. The distribution trend of the springback rate is basically consistent with the experimental results.

**Figure 18:**
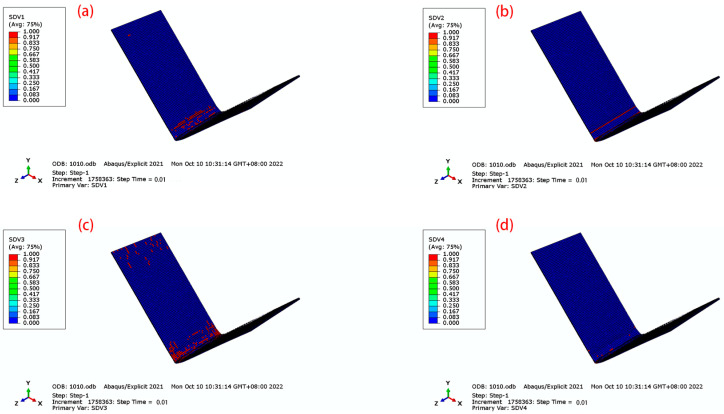
Damage of CFRP for 45 orthotropic. (**a**) SDV1; (**b**) SDV2; (**c**) SDV3; (**d**) SDV4.

**Figure 19:**
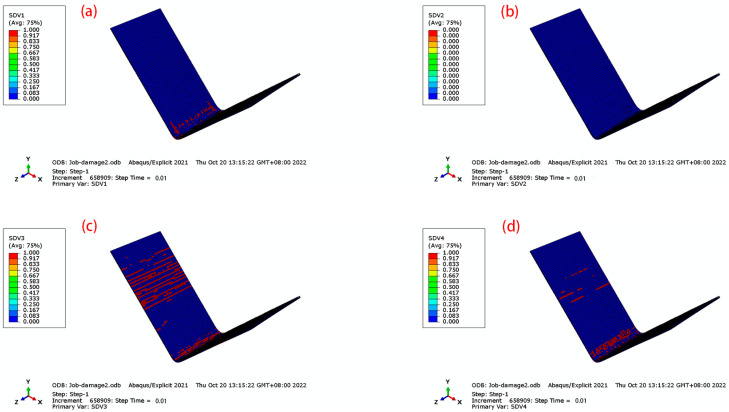
Damage to CFRP for 90 unidirectional. (**a**) SDV1; (**b**) SDV2; (**c**) SDV3; (**d**) SDV4.

For the effects of different lay-ups on the damage, the 45° orthotropic and 90° unidirectional lay-ups were selected, as shown in Figure 18 and Figure 19. For the fiber damage of SDV1 and SDV2, the laminate with 90° lay-up has less damage, which is due to the fact that 90° fibers carry less load along the fiber direction during the bending process. For the matrix damage of SDV3 and SDV4, the difference between 45° orthotropic and 90° unidirectional lay-ups is great. The 90° fibers have a weak pressure-bearing capacity, and during the loading process, the resin matrix was loaded along the fiber direction. The 45° orthotropic has a strong compressive capacity, and the damage to the matrix is less.

**Figure 20:**
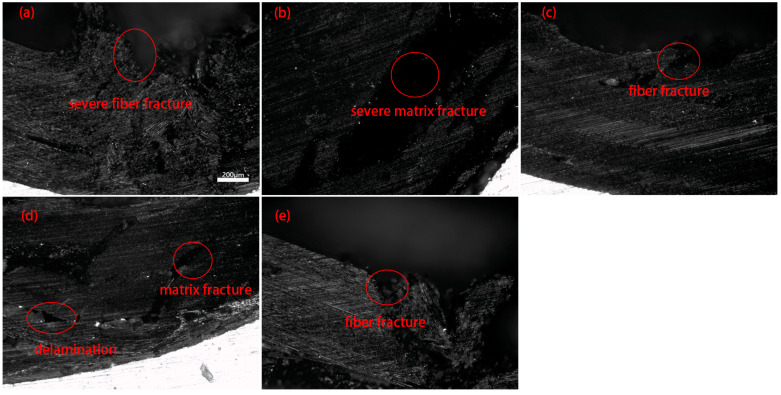
Micromorphology of different lay-ups. (**a**) 0 unidirectional; (**b**) 90 unidirectional; (**c**) 45 orthotropic; (**d**) 90 orthotropic; (**e**) 0 orthotropic.

Microscopic observation of the molding area is shown in Figure 20. Unidirectional fibers can lead to severe damage behavior, such as fiber fracture, for the 0° unidirectional lay-up and matrix fracture for the 90° unidirectional lay-up. For the laminates with 45° orthotropic lay-up, partial fiber breakage of the outer layer is visible, which is relatively less damaged. For the laminate with the 90° orthotropic lay-up, the same matrix fracture is seen in the 90° fibers of the core layer. In addition, fiber delamination is seen at the junctions of the 0° and 90° fibers. The appropriate lay-up method can reduce the occurrence of damage, which verifies the accuracy of damage prediction using the finite element method.

## 5. Conclusions

The forming accuracy of 7075-T6 aluminum alloy/CFRP laminate was investigated using experimental and numerical analysis methods. The results of the single-lap shear test showed that the suitable curing conditions are 130 °C for 45 min. No interface debonding between Al/CFRP was observed under this curing condition. In the V-shaped bending experiments, the 45° orthotropic lay-up had the lowest springback rate of 1.11%, which was 80% lower compared to the 90° unidirectional lay-up. Increasing the thickness of the metal layer reduced the springback. The springback of CFRP/Al laminates was not sensitive to the forming speed. The simulations of spingback were consistent with the experimental results, and by subroutine Vumat and microscopic analysis, it can be stated that the appropriate lay-ups could effectively reduce the damage to the fibers and the matrix.

## Figures and Tables

**Table 1:** Mechanical properties of T700.

Density (kg/cm^3^)	E1 (GPa)	E2 (GPa)	μ12	G12 (GPa)	G13 (GPa)	G23 (GPa)
1560	130	8	0.28	4.5	4.5	3.6

**Table 2:** Mechanical properties of aluminum alloy 7075-T6.

Density (kg/cm^3^)	Tensile Strength (GPa)	Yield Strength (GPa)	E (GPa)	Elongation %
2800	0.55	0.48	64	10

**Table 3:** Elastic modulus of CFRP.

Lay-Up	0 Unidirectional	90 Unidirectional	90 Orthotropic	0 Orthotropic	45 Orthotropic
E (GPa)	130	8	48.67	89.33	16

**Table 4:** Elastic modulus of CFRP/Al.

Lay-Up	0 Unidirectional	90 Unidirectional	90 Orthotropic	0 Orthotropic	45 Orthotropic
E (GPa)	79.28	51.12	60.47	69.82	66.76

**Table 5:** Mechanical properties of 7075 aluminum with 130 °C-45 min aging.

E (GPa)	Tensile Strength (GPa)	Yield Strength (GPa)	Density (kg/cm^3^)	Elongation %
69	0.37	0.27	2800	20

## Data Availability

Not applicable.
